# Back From the Past: A Case of Transjugular Intrahepatic Shunt-Induced Hemolysis?

**DOI:** 10.7759/cureus.94692

**Published:** 2025-10-16

**Authors:** Genan Arman, Ahmad El-Sheikh, Hajar El Amri, Sreeja Sompalli

**Affiliations:** 1 Internal Medicine, Southern Illinois University School of Medicine, Springfield, USA; 2 Pulmonary and Critical Care Medicine, Southern Illinois University School of Medicine, Springfield, USA; 3 Internal Medicine, Atlanticare Regional Medical Center, Atlantic City, USA

**Keywords:** coomb's negative, decompensated alcoholic liver disease, intravascular hemolysis, liver cirhosis, transjugular, transjugular intrahepatic portosytemic shunt, viatorr stent

## Abstract

Transjugular intrahepatic portosystemic shunt (TIPS)-induced hemolytic anemia was a known complication of the previously used bare metal wall stent, but it is rarely associated with the modern Viatorr stent. It is important to consider this diagnosis in specific patients, as replacement of the stent results in the resolution of the hemolysis. A 49-year-old female with alcoholic liver cirrhosis, classified as Child Pugh-C, and esophageal varices, who underwent emergent TIPS placement six weeks prior, presented with shortness of breath and acute anemia. All sources of active bleeding were excluded. Lab work and peripheral blood smear were consistent with hemolytic anemia. Hemolytic anemia was attributed to TIPS-induced hemolysis, and the patient was referred for a liver transplant. Unfortunately, she passed away before the transplant could be performed, most likely due to the persistent, unresolved anemia. This report highlights the importance of considering TIPS-induced hemolysis in the differential diagnosis of anemia, even in patients who have received the new Viatorr stent. Ruling out other causes is necessary. Replacement of the stent results in the resolution of the hemolysis. Early recognition is crucial for effective management.

## Introduction

The transjugular intrahepatic portosystemic shunt (TIPS) procedure is a minimally invasive technique employed to address complications arising from portal hypertension, often observed in patients with advanced liver disease. By establishing a shunt between the portal vein and the hepatic vein, TIPS effectively lowers the elevated pressure within the portal vein system, thereby mitigating conditions like variceal bleeding and refractory ascites [1]. Historically, hemolysis induced by TIPS was more common with bare metal stents, estimated to occur in approximately 10% of patients [2]. While the precise mechanism linking TIPS to hemolytic anemia remains unclear, it has been linked to factors such as increased shear stress within the newly formed shunt, changes in blood flow, and pre-existing liver disease [1]. At the start of the 20th century, polytetrafluoroethylene-covered stents became the standard due to their improved patency. However, a small 2-cm segment of these stents remains base, which still allows for the possibility of hemolysis [3].

## Case presentation

**Figure 1:**
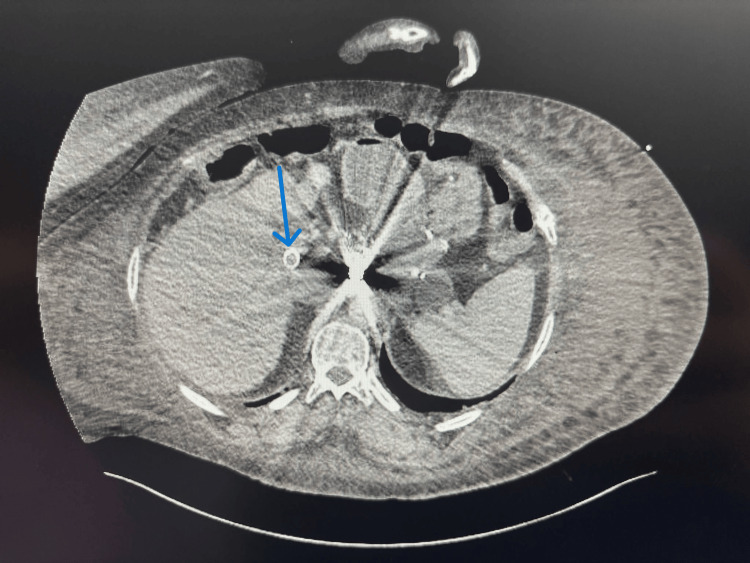
CTA abdomen - coronal view featuring patent TIPS CTA: computed tomography angiogram; TIPS: transjugular intrahepatic portosystemic shunt

**Figure 2:**
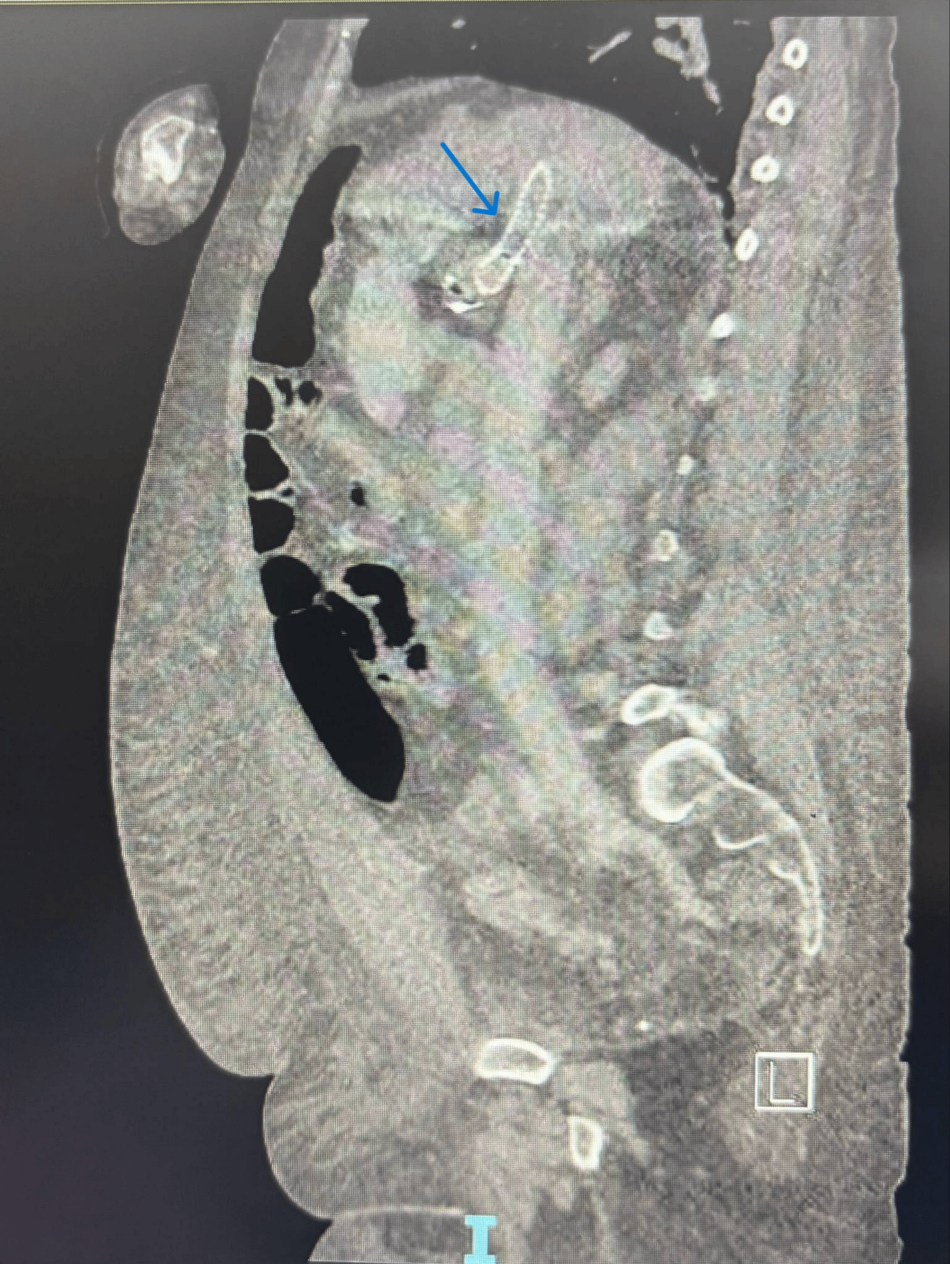
CT chest, abdomen, and pelvis - sagittal view featuring patent TIPS CT: computed tomography; TIPS: transjugular intrahepatic portosystemic shunt

A 49-year-old female with a history of alcoholic liver cirrhosis, classified as Child-Pugh C, and esophageal varices, for which she had undergone an emergent TIPS placement two months prior, presented to the emergency department with a five-day history of hematochezia, accompanied by shortness of breath and worsening lower extremity edema. In the emergency department, she was initially stable, and a CT angiogram (CTA) was negative for any source of bleeding and showed patent TIPS (Figures 1, 2).

Shortly after, the patient developed hemorrhagic shock and a suspected brisk upper gastrointestinal bleed. She was subsequently admitted to the ICU. Her vital signs included a mean arterial pressure of 55 mmHg, a heart rate of 105 beats/min, and a hemoglobin level of 4.1 g/dL. She was started on a massive transfusion protocol, vasopressors, proton pump inhibitors (PPI), octreotide, and ceftriaxone.

**Figure 3:**
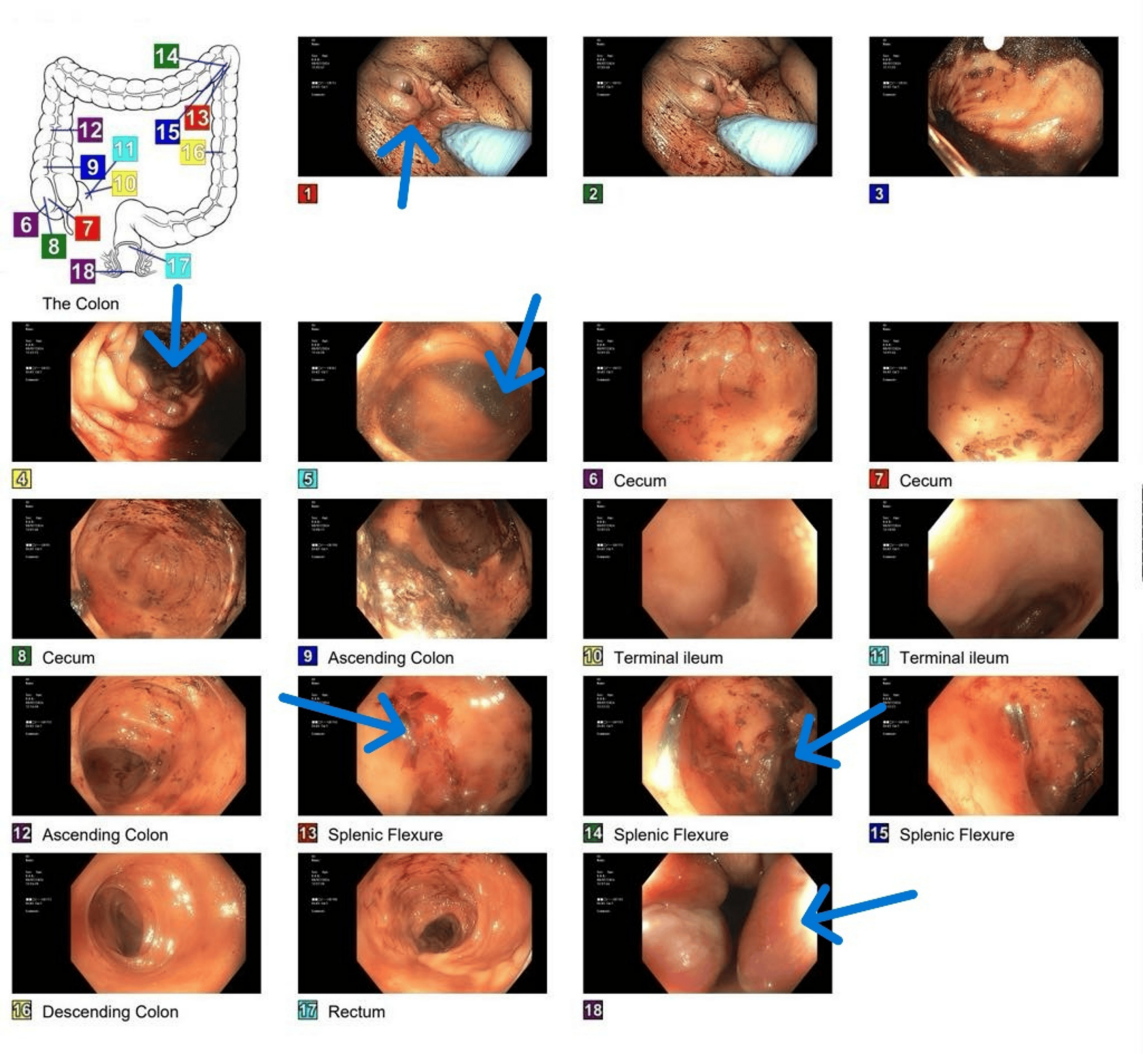
Colonoscopy findings Nonbleeding internal hemorrhoids (1, 18), a single nonbleeding erosion in the splenic flexure (13, 14), friability of the colon (4), and blood in the colon (5)

Gastroenterology was consulted, and a technetium-99m scan was done, which showed small bleeding in the stomach; however, a subsequent esophagogastroduodenoscopy (EGD) showed no evidence of bleeding esophageal or gastric varices. A bedside colonoscopy revealed blood in the colon, an erosion of the splenic flexure, and nonbleeding internal hemorrhoids, but otherwise no source of bleeding (Figure 3). 

Despite a reduction of blood clots in her stools, the patient continued to require transfusions due to persistently declining hemoglobin levels. Her laboratory results were indicative of hemolytic anemia (Tables 1, 2). She also had a total bilirubin level of 9.1 mg/dL. A Coombs test was negative, and a peripheral blood smear showed two schistocytes/high power field. A coagulation panel as below (Table 2) ruled out disseminated intravascular coagulation (DIC). The absence of fever and altered mental status, along with stable thrombocytopenia and kidney function, ruled out thrombotic thrombocytopenic purpura (TTP) and hemolytic uremic syndrome (HUS).

**Table 1:** Coagulation, liver, and renal function labs PT: prothrombin time; INR: international normalized ratio; aPTT: activated partial thromboplastin time; BUN: blood urea nitrogen; AST: aspartate aminotransferase; ALT: alanine aminotransferase; LDH: lactate dehydrogenase

Lab test	Patient value	Reference range
PT, seconds	19.2	11.6 – 14.5
INR	1.6	0.9 – 1.1
aPTT, seconds	35.7	22.9 – 35.1
Fibrinogen, mg/dL	210	200 – 400
Fibrin degradation products, µg/mL	10	<10
BUN, mg/dL	23	7 – 20
Creatinine, mg/dL	1.2	0.6 – 1.3
AST, U/L	73	10 – 40
ALT, U/L	23	7 – 56
Total bilirubin, mg/dL	9.1	0.2 – 1.2
Direct bilirubin, mg/dL	3.0	0 – 0.3
LDH, U/L	198	140 – 280
Haptoglobin, mg/dL	<30	30 – 200

**Table 2:** CBC with differential cell count CBC: complete blood count; RBC: red blood cell; MCV: mean corpuscular volume; MCH: mean corpuscular hemoglobin; MCHC: mean corpuscular hemoglobin concentration; RDW: red cell distribution width; WBC: white blood cells; MPV: mean platelet volume

Lab test	Patient value	Reference range
Hemoglobin, g/dL	4.1	12 – 16
Hematocrit, %	12	37 – 47
RBC, x10⁶/cumm	1.16	4.2 – 5.4
MCV, fL	101	81 – 94
MCH, pg	35.8	27.5 – 33.2
MCHC, g/dL	35.3	31 – 36
RDW, %	22.7	11.7 – 15.5
WBC, x10³/cumm	10.2	3.4 – 9.4
Dimorphic RBCs	Present	—
Schistocytes	Few	—
Pocked RBCs	Few	—
Elliptocytes	Moderate	—
Burr cells	Moderate	—
Teardrop cells	Few	—
Acanthocytes	<5%	—
Platelets, x10³/µL	143	140 – 410
MPV, fL	7.3	—

A TIPS venogram showed a patent TIPS with a porto-systemic shunt gradient of 8 mmHg. The patient’s hemolysis spontaneously resolved within two weeks of presentation but reoccurred a month later. She was referred for a liver transplant, but unfortunately, her medical status declined, requiring ventilatory and pressor support. A shared decision was made by consulting the family to withdraw pressure support, and the patient passed away shortly after.

## Discussion

Hemolytic anemia was a well-known complication of TIPS in the past, in an era before switching from the bare metal Wallstent to the new Viatorr stent, with a reported incidence of 10% [2]. It was marked by an increase in reticulocytes, elevated indirect bilirubin, reduced haptoglobin, and higher lactate dehydrogenase levels with or without schistocytes. Identifying hemolysis as a complication of TIPS requires ruling out other causes of anemia, such as gastrointestinal bleeding, decreased red blood cell production due to chronic disease, alcohol toxicity, and nutritional deficiencies [1].

Hemolysis with the bare metal Wallstent was related to erythrocytes colliding with the un-endothelialized bare wire mesh, particularly near the free hepatic and portal venous ends of the stent [3], also known as naked stent syndrome [3]. However, with the adoption of the Viatorr stent graft, TIPS-associated hemolytic anemia became rare. This could be explained by the fact that most of the Viatorr stent is lined with smooth expanded polytetrafluoroethylene (ePTFE), which reduces red cell collisions with bare metal interstices [4]. A few cases of this phenomenon occurring with a Viatorr stent graft have been reported [1,5,6]. Attributing this to the 2-cm bare metal segment in the Viatorr stents, which is kept allowing portal perfusion, this short segment may theoretically introduce a site for RBC injury [1].

Although TIPS-associated hemolytic anemia may seem similar to hemolytic anemia related to other implantable devices, it has some interesting differences. Flow velocities greater than 1,000-2,000 cm/s are usually required for shear stress hemolysis to occur [7]. Velocities within TIPS are typically far less [8]. In contrast to other implanted device-related hemolysis, schistocytes are usually absent on the peripheral smear and are not required for the diagnosis. The primary mechanism of RBC injury is thought to be collision sufficient enough to induce erythrophagocytosis, and subsequent removal of the damaged RBCs by the spleen or circulating phagocytes [2,9]. Cirrhosis and hypersplenism often cause intrinsic structural abnormalities and shorter erythrocyte half-lives. All of which are presumed to enhance the susceptibility for injury and subsequent erythrophagocytosis [2].

Additionally, TIPS hemolysis is self-limiting, in contrast to the chronic hemolysis seen in artificial heart valves; most hemolysis cases resolve on their own in less than 12 weeks [7]. However, some previously documented cases showed improvement only after receiving a liver transplant [7]. This spontaneous remission timeline is thought to coincide with the neointima creation inside the stent, which lessens the chance of injury from red blood cells colliding with the interstices in the stent [7].

## Conclusions

This report described a diagnostic dilemma where multiple causes of anemia may coincide. In our patient, anemia was hemolytic but compounded by minor intermittent gastrointestinal bleeding. This report also highlights the importance of considering TIPS-induced hemolysis in the differential diagnosis of anemia in patients with recent stent placement, even with the new Viatorr stent. Ruling out other causes of hemolytic anemia is necessary, but the presence of schistocytes is not required for the diagnosis. Early detection and treatment with replacement of the stent results in the resolution of the hemolysis and may be necessary in some cases. Early recognition is crucial to guide effective management. It is important to consider hemolysis in the differential diagnosis even in patients with the newer Viatorr stents. However, this entity remains relatively unexplored with respect to newer stents. There is a need for further case reports or case series to consolidate the current knowledge on this rare complication.
